# Supplementary material for: A Critical View of the Application of the APEX Software (Aqueous Photochemistry of Environmentally-Occurring Xenobiotics) to Predict Photoreaction Kinetics in Surface Freshwaters
Source: Molecules. 2019 Dec 18;25(1):9. doi: 10.3390/molecules25010009 (PMC7017383; doi:10.3390/molecules25010009)
Supplement: Supplementary file 1 [file molecules-25-00009-s001.zip › 3.2.4_gcc-4.4.0.zip!/3.2.4_gcc-4.4.0/3.2.4_gcc-4.4.0/doc/octave/html/interpreter/Rearranging-Matrices.html]

Rearranging Matrices - Untitled


Next: Applying a Function to an Array,
Previous: Finding Elements and Checking Conditions,
Up: Matrix Manipulation


---

### 16.2 Rearranging Matrices

— Function File: **fliplr** (x)  
> Return a copy of x with the order of the columns reversed. For
> example,
>
> ```
>           fliplr ([1, 2; 3, 4])
>                =>  2  1
>                    4  3
> ```
>
> Note that `fliplr` only work with 2-D arrays. To flip N-d arrays
> use `flipdim` instead.
>
>
>
> **See also:** flipud, flipdim, rot90, rotdim.

— Function File: **flipud** (x)  
> Return a copy of x with the order of the rows reversed. For
> example,
>
> ```
>           flipud ([1, 2; 3, 4])
>                =>  3  4
>                    1  2
> ```
>
> Due to the difficulty of defining which axis about which to flip the
> matrix `flipud` only work with 2-d arrays. To flip N-d arrays
> use `flipdim` instead.
>
>
>
> **See also:** fliplr, flipdim, rot90, rotdim.

— Function File: **flipdim** (x, dim)  
> Return a copy of x flipped about the dimension dim.
> For example
>
> ```
>           flipdim ([1, 2; 3, 4], 2)
>                =>  2  1
>                    4  3
> ```
>
>
>
>
> **See also:** fliplr, flipud, rot90, rotdim.

— Function File: **rot90** (x, n)  
> Return a copy of x with the elements rotated counterclockwise in
> 90-degree increments. The second argument is optional, and specifies
> how many 90-degree rotations are to be applied (the default value is 1).
> Negative values of n rotate the matrix in a clockwise direction.
> For example,
>
> ```
>           rot90 ([1, 2; 3, 4], -1)
>                =>  3  1
>                    4  2
> ```
>
> rotates the given matrix clockwise by 90 degrees. The following are all
> equivalent statements:
>
> ```
>           rot90 ([1, 2; 3, 4], -1)
>           rot90 ([1, 2; 3, 4], 3)
>           rot90 ([1, 2; 3, 4], 7)
> ```
>
> Due to the difficulty of defining an axis about which to rotate the
> matrix `rot90` only work with 2-D arrays. To rotate N-d arrays
> use `rotdim` instead.
>
>
>
> **See also:** rotdim, flipud, fliplr, flipdim.

— Function File: **rotdim** (x, n, plane)  
> Return a copy of x with the elements rotated counterclockwise in
> 90-degree increments. The second argument is optional, and specifies
> how many 90-degree rotations are to be applied (the default value is 1).
> The third argument is also optional and defines the plane of the
> rotation. As such plane is a two element vector containing two
> different valid dimensions of the matrix. If plane is not given
> Then the first two non-singleton dimensions are used.
>
> Negative values of n rotate the matrix in a clockwise direction.
> For example,
>
> ```
>           rotdim ([1, 2; 3, 4], -1, [1, 2])
>                =>  3  1
>                    4  2
> ```
>
> rotates the given matrix clockwise by 90 degrees. The following are all
> equivalent statements:
>
> ```
>           rotdim ([1, 2; 3, 4], -1, [1, 2])
>           rotdim ([1, 2; 3, 4], 3, [1, 2])
>           rotdim ([1, 2; 3, 4], 7, [1, 2])
> ```
>
>
>
>
> **See also:** rot90, flipud, fliplr, flipdim.

— Built-in Function: **cat** (dim, array1, array2, ..., arrayN)  
> Return the concatenation of N-d array objects, array1,
> array2, ..., arrayN along dimension dim.
>
> ```
>           A = ones (2, 2);
>           B = zeros (2, 2);
>           cat (2, A, B)
>           => ans =
>           
>                1 1 0 0
>                1 1 0 0
> ```
>
> Alternatively, we can concatenate A and B along the
> second dimension the following way:
>
> ```
>           [A, B].
> ```
>
> dim can be larger than the dimensions of the N-d array objects
> and the result will thus have dim dimensions as the
> following example shows:
>
> ```
>           cat (4, ones(2, 2), zeros (2, 2))
>           => ans =
>           
>              ans(:,:,1,1) =
>           
>                1 1
>                1 1
>           
>              ans(:,:,1,2) =
>                0 0
>                0 0
> ```
>
>
>
>
> **See also:** horzcat, vertcat.

— Built-in Function: **horzcat** (array1, array2, ..., arrayN)  
> Return the horizontal concatenation of N-d array objects, array1,
> array2, ..., arrayN along dimension 2.
>
>
>
> **See also:** cat, vertcat.

— Built-in Function: **vertcat** (array1, array2, ..., arrayN)  
> Return the vertical concatenation of N-d array objects, array1,
> array2, ..., arrayN along dimension 1.
>
>
>
> **See also:** cat, horzcat.

— Built-in Function: **permute** (a, perm)  
> Return the generalized transpose for an N-d array object a.
> The permutation vector perm must contain the elements
> `1:ndims(a)` (in any order, but each element must appear just once).
>
>
>
> **See also:** ipermute.

— Built-in Function: **ipermute** (a, iperm)  
> The inverse of the `permute` function. The expression
>
> ```
>           ipermute (permute (a, perm), perm)
> ```
>
> returns the original array a.
>
>
>
> **See also:** permute.

— Built-in Function: **reshape** (a, m, n, ...)  
— Built-in Function: **reshape** (a, size)  
> Return a matrix with the given dimensions whose elements are taken
> from the matrix a. The elements of the matrix are accessed in
> column-major order (like Fortran arrays are stored).
>
> For example,
>
> ```
>           reshape ([1, 2, 3, 4], 2, 2)
>                =>  1  3
>                    2  4
> ```
>
> Note that the total number of elements in the original
> matrix must match the total number of elements in the new matrix.
>
> A single dimension of the return matrix can be unknown and is flagged
> by an empty argument.

— Built-in Function: **resize** (x, m)  
— Built-in Function: **resize** (x, m, n)  
— Built-in Function: **resize** (x, m, n, ...)  
> Resize x cutting off elements as necessary.
>
> In the result, element with certain indices is equal to the corresponding
> element of x if the indices are within the bounds of x;
> otherwise, the element is set to zero.
>
> In other words, the statement
>
> ```
>             y = resize (x, dv);
> ```
>
> is equivalent to the following code:
>
> ```
>             y = zeros (dv, class (x));
>             sz = min (dv, size (x));
>             for i = 1:length (sz), idx{i} = 1:sz(i); endfor
>             y(idx{:}) = x(idx{:});
> ```
>
> but is performed more efficiently.
>
> If only m is supplied and it is a scalar, the dimension of the
> result is m-by-m. If m is a vector, then the
> dimensions of the result are given by the elements of m.
> If both m and n are scalars, then the dimensions of
> the result are m-by-n.
>
> An object can be resized to more dimensions than it has;
> in such case the missing dimensions are assumed to be 1.
> Resizing an object to fewer dimensions is not possible.
>
>
>
> **See also:** reshape, postpad.

— Function File: y = **circshift** (x, n)  
> Circularly shifts the values of the array x. n must be
> a vector of integers no longer than the number of dimensions in
> x. The values of n can be either positive or negative,
> which determines the direction in which the values or x are
> shifted. If an element of n is zero, then the corresponding
> dimension of x will not be shifted. For example
>
> ```
>           x = [1, 2, 3; 4, 5, 6; 7, 8, 9];
>           circshift (x, 1)
>           =>  7, 8, 9
>               1, 2, 3
>               4, 5, 6
>           circshift (x, -2)
>           =>  7, 8, 9
>               1, 2, 3
>               4, 5, 6
>           circshift (x, [0,1])
>           =>  3, 1, 2
>               6, 4, 5
>               9, 7, 8
> ```
>
>
>
>
> **See also:** permute, ipermute, shiftdim.

— Function File: y = **shiftdim** (x, n)  
— Function File: [y, ns] = **shiftdim** (x)  
> Shifts the dimension of x by n, where n must be
> an integer scalar. When n is positive, the dimensions of
> x are shifted to the left, with the leading dimensions
> circulated to the end. If n is negative, then the dimensions
> of x are shifted to the right, with n leading singleton
> dimensions added.
>
> Called with a single argument, `shiftdim`, removes the leading
> singleton dimensions, returning the number of dimensions removed
> in the second output argument ns.
>
> For example
>
> ```
>           x = ones (1, 2, 3);
>           size (shiftdim (x, -1))
>                => [1, 1, 2, 3]
>           size (shiftdim (x, 1))
>                => [2, 3]
>           [b, ns] = shiftdim (x);
>                => b =  [1, 1, 1; 1, 1, 1]
>                => ns = 1
> ```
>
>
>
>
> **See also:** reshape, permute, ipermute, circshift, squeeze.

— Function File: **shift** (x, b)  
— Function File: **shift** (x, b, dim)  
> If x is a vector, perform a circular shift of length b of
> the elements of x.
>
> If x is a matrix, do the same for each column of x.
> If the optional dim argument is given, operate along this
> dimension

— Loadable Function: [s, i] = **sort** (x)  
— Loadable Function: [s, i] = **sort** (x, dim)  
— Loadable Function: [s, i] = **sort** (x, mode)  
— Loadable Function: [s, i] = **sort** (x, dim, mode)  
> Return a copy of x with the elements arranged in increasing
> order. For matrices, `sort` orders the elements in each column.
>
> For example,
>
> ```
>           sort ([1, 2; 2, 3; 3, 1])
>                =>  1  1
>                    2  2
>                    3  3
> ```
>
> The `sort` function may also be used to produce a matrix
> containing the original row indices of the elements in the sorted
> matrix. For example,
>
> ```
>           [s, i] = sort ([1, 2; 2, 3; 3, 1])
>                => s = 1  1
>                       2  2
>                       3  3
>                => i = 1  3
>                       2  1
>                       3  2
> ```
>
> If the optional argument dim is given, then the matrix is sorted
> along the dimension defined by dim. The optional argument `mode`
> defines the order in which the values will be sorted. Valid values of
> `mode` are `ascend' or `descend'.
>
> For equal elements, the indices are such that the equal elements are listed
> in the order that appeared in the original list.
>
> The `sort` function may also be used to sort strings and cell arrays
> of strings, in which case the dictionary order of the strings is used.
>
> The algorithm used in `sort` is optimized for the sorting of partially
> ordered lists.

— Function File: **sortrows** (a, c)  
> Sort the rows of the matrix a according to the order of the
> columns specified in c. If c is omitted, a
> lexicographical sort is used. By default ascending order is used
> however if elements of c are negative then the corresponding
> column is sorted in descending order.

— Built-in Function: **issorted** (a, rows)  
> Returns true if the array is sorted, ascending or descending.
> NaNs are treated as by `sort`. If rows is supplied and
> has the value "rows", checks whether the array is sorted by rows
> as if output by `sortrows` (with no options).
>
> This function does not yet support sparse matrices.
>
>
>
> **See also:** sortrows, sort.

Since the `sort` function does not allow sort keys to be specified,
it can't be used to order the rows of a matrix according to the values
of the elements in various columns^1^
in a single call. Using the second output, however, it is possible to
sort all rows based on the values in a given column. Here's an example
that sorts the rows of a matrix based on the values in the second
column.

```
     a = [1, 2; 2, 3; 3, 1];
     [s, i] = sort (a (:, 2));
     a (i, :)
          =>  3  1
              1  2
              2  3
```

— Function File: **tril** (a, k)  
— Function File: **triu** (a, k)  
> Return a new matrix formed by extracting the lower (`tril`)
> or upper (`triu`) triangular part of the matrix a, and
> setting all other elements to zero. The second argument is optional,
> and specifies how many diagonals above or below the main diagonal should
> also be set to zero.
>
> The default value of k is zero, so that `triu` and
> `tril` normally include the main diagonal as part of the result
> matrix.
>
> If the value of k is negative, additional elements above (for
> `tril`) or below (for `triu`) the main diagonal are also
> selected.
>
> The absolute value of k must not be greater than the number of
> sub- or super-diagonals.
>
> For example,
>
> ```
>           tril (ones (3), -1)
>                =>  0  0  0
>                    1  0  0
>                    1  1  0
> ```
>
> and
>
> ```
>           tril (ones (3), 1)
>                =>  1  1  0
>                    1  1  1
>                    1  1  1
> ```
>
>
>
>
> **See also:** triu, diag.

— Function File: **vec** (x)  
> Return the vector obtained by stacking the columns of the matrix x
> one above the other.

— Function File: **vech** (x)  
> Return the vector obtained by eliminating all supradiagonal elements of
> the square matrix x and stacking the result one column above the
> other.

— Function File: **prepad** (x, l, c)  
— Function File: **prepad** (x, l, c, dim)  
> Prepend (append) the scalar value c to the vector x
> until it is of length l. If the third argument is not
> supplied, a value of 0 is used.
>
> If `length (`x`) >` l, elements from the beginning (end) of
> x are removed until a vector of length l is obtained.
>
> If x is a matrix, elements are prepended or removed from each row.
>
> If the optional dim argument is given, then operate along this
> dimension.
>
>
>
> **See also:** postpad.

— Built-in Function: **diag** (v, k)  
> Return a diagonal matrix with vector v on diagonal k. The
> second argument is optional. If it is positive, the vector is placed on
> the k-th super-diagonal. If it is negative, it is placed on the
> -k-th sub-diagonal. The default value of k is 0, and the
> vector is placed on the main diagonal. For example,
>
> ```
>           diag ([1, 2, 3], 1)
>                =>  0  1  0  0
>                    0  0  2  0
>                    0  0  0  3
>                    0  0  0  0
> ```
>
> Given a matrix argument, instead of a vector, `diag` extracts the
> k-th diagonal of the matrix.

— Function File: **blkdiag** (a, b, c, ...)  
> Build a block diagonal matrix from a, b, c, ....
> All the arguments must be numeric and are two-dimensional matrices or
> scalars.
>
>
>
> **See also:** diag, horzcat, vertcat.

---

#### Footnotes

[1] For example, to first sort
based on the values in column 1, and then, for any values that are
repeated in column 1, sort based on the values found in column 2, etc.

---
